# Differentiating Protein-Coding and Noncoding RNA: Challenges and Ambiguities

**DOI:** 10.1371/journal.pcbi.1000176

**Published:** 2008-11-28

**Authors:** Marcel E. Dinger, Ken C. Pang, Tim R. Mercer, John S. Mattick

**Affiliations:** 1 ARC Special Research Centre for Functional and Applied Genomics, Institute for Molecular Bioscience, University of Queensland, St Lucia, Australia; 2 T cell Laboratory, Ludwig Institute for Cancer Research, Melbourne Centre for Clinical Sciences, Austin Health, Heidelberg, Australia; National Center for Biotechnology Information (NCBI), United States of America

## Abstract

The assumption that RNA can be readily classified into either protein-coding or non-protein–coding categories has pervaded biology for close to 50 years. Until recently, discrimination between these two categories was relatively straightforward: most transcripts were clearly identifiable as protein-coding messenger RNAs (mRNAs), and readily distinguished from the small number of well-characterized non-protein–coding RNAs (ncRNAs), such as transfer, ribosomal, and spliceosomal RNAs. Recent genome-wide studies have revealed the existence of thousands of noncoding transcripts, whose function and significance are unclear. The discovery of this hidden transcriptome and the implicit challenge it presents to our understanding of the expression and regulation of genetic information has made the need to distinguish between mRNAs and ncRNAs both more pressing and more complicated. In this Review, we consider the diverse strategies employed to discriminate between protein-coding and noncoding transcripts and the fundamental difficulties that are inherent in what may superficially appear to be a simple problem. Misannotations can also run in both directions: some ncRNAs may actually encode peptides, and some of those currently thought to do so may not. Moreover, recent studies have shown that some RNAs can function both as mRNAs and intrinsically as functional ncRNAs, which may be a relatively widespread phenomenon. We conclude that it is difficult to annotate an RNA unequivocally as protein-coding or noncoding, with overlapping protein-coding and noncoding transcripts further confounding this distinction. In addition, the finding that some transcripts can function both intrinsically at the RNA level and to encode proteins suggests a false dichotomy between mRNAs and ncRNAs. Therefore, the functionality of any transcript at the RNA level should not be discounted.

## Introduction

Numerous studies have demonstrated that the true catalog of RNAs encoded within the genome (the “transcriptome”) is more extensive and complex than previously thought (reviewed in [1]–[3]). In humans and mice, for instance, it has become apparent that the vast majority of the genome is transcribed, often in intricate networks of overlapping sense and antisense transcripts, many of which are alternatively spliced [1], [4]–[8]. However, mRNAs account for only ∼2.3% of the human genome [1],[9], and therefore the vast majority of this unexpected transcription, sometimes referred to as “dark matter” [10],[11], appears to be non-protein–coding.

Unsurprisingly, a great deal of attention is now focused on the noncoding transcriptome. Dominating this field of inquiry has been the discovery of thousands of small RNAs (<200 nt in length). Many of these have since been classified into novel categories (e.g., microRNAs, PIWI-associated RNAs, and endogenous small interfering RNAs) on the basis of function, length, biogenesis, structural/sequence features, and protein-binding partners (reviewed in [12]). Interestingly, however, long ncRNAs (>200 nt) appear to comprise the largest portion of the mammalian noncoding transcriptome. Tiling array studies of the human genome, for instance, revealed that the majority of transcription occurs as long ncRNAs [13], some of which may be precursors for smaller RNAs, but many of which are detected as relatively stable polyadenylated and non-polyadenylated transcripts [8],[14].

The biological significance of these long ncRNAs is controversial. Despite an increasing number of long ncRNAs having been shown to fulfill a diverse range of regulatory roles (reviewed in [15],[16]), the functions of the vast majority remain unknown and untested. And while this is also true of small RNAs to some extent, long ncRNAs—unlike their smaller counterparts—lack obvious features to allow a priori functional categorization or prediction. Furthermore, the exact prevalence of long ncRNAs remains subject to significant interpretation and debate. For instance, the FANTOM and H-Invitational consortiums annotated comprehensive full-length cDNA collections in mouse and human, respectively [5],[17]. Despite using similar methods of cDNA library construction, the two groups came up with very different prevalence estimates for long ncRNAs within the mammalian genome: in mouse, 33% of transcripts (34,030/102,281) were annotated as noncoding; by comparison, only 7% of human transcripts (1,377/21,037) were identified as ncRNAs. That such divergent estimates exist highlights how difficult it has become to discriminate between long ncRNAs and mRNAs. To better understand the nature of these challenges, the approaches used to distinguish noncoding from protein-coding are considered below.

## Strategies to Discriminate between ncRNAs and mRNAs

### 

#### Open reading frame length

**Figure 1:**
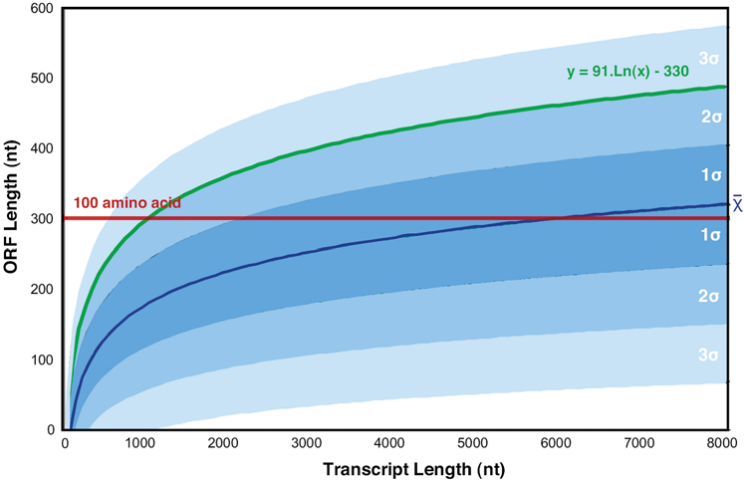
Incidence of open reading frames (ORFs) in randomly generated transcripts of increasing length. Twenty thousand transcripts of varying length and random nucleotide composition were computationally generated and scanned for ORFs. The maximum ORF and transcript lengths were plotted and fitted to a logarithmic curve. The shaded regions represent incidences of randomly occurring ORFs at 1, 2, or 3 standard deviations from the mean. The red line indicates the 300 nt ORF threshold that is commonly used to distinguish protein-coding genes in transcript classification pipelines. Therefore, this plot illustrates that for transcripts longer than ∼1000 bp, such a threshold may define transcripts as protein-coding that would be expected to occur by chance. The function y = 91.Ln(x)−330, which approximates random ORF incidence according to transcript length at two standard deviations above the mean (i.e., 95% confidence interval, indicated in green), could be used to discriminate noncoding from protein-coding transcripts in a transcript-length–dependent manner.

One of the most fundamental criteria used to distinguish long ncRNAs from mRNAs is ORF length. Since short putative ORFs can be expected to occur by chance within long noncoding sequences, minimum ORF cutoffs are usually applied to reduce the likelihood of falsely categorizing ncRNAs as mRNAs. For instance, the FANTOM consortium originally used a cutoff of 300 nt (100 codons) to help identify putative mRNAs [18]. This somewhat arbitrary threshold is consistent with the observation that >95% of proteins in public databases such as Swiss-Prot and the International Protein Index are >100 aa in length [19], and has subsequently been shown to display a high level of concordance with more sophisticated discrimination methods [20]. This length is also approximately two standard deviations above the average length of ORFs in a one kilobase random sequence (Figure 1).

Using putative ORF length alone, although straightforward to apply across large datasets, is problematic for various reasons. First, bona fide long ncRNAs will by chance contain putative ORFs that are quite long. For instance, *H19*, *Xist*, *Mirg*, *Gtl2*, and *KcnqOT1* all have putative ORFs >100 codons, but have been characterized as functional ncRNAs [15]. Applying a traditional ORF cutoff of 300 nt will therefore misclassify many ncRNAs as mRNAs, and this is especially true for very long ncRNAs, as illustrated in Figure 1. For example, murine *Xist* is ∼15 Kb in size [21] and contains a putative ORF of 298 aa, which led to the erroneous conclusion that it was a protein-coding gene when first discovered [22]. Second, with a cutoff of 300 nt, proteins <100 aa in size may also be incorrectly classified as ncRNAs. The potential scale of such errors is significant, given recent estimates that the mammalian proteome contains ∼3,700 proteins below this size [19]. To minimize such errors, ORF length cutoffs can be reduced, which is exactly what the H-Invitational consortium did in applying a threshold of 60 nt (20 codons) in their annotation pipeline [17]. However, such a low threshold will falsely underestimate the number of ncRNAs, which probably explains to a large extent why the numbers of H-Invitational ncRNAs are so small. Finally, it is notable that even at very low cutoffs, some atypical proteins will still be missed. The *tarsal-less* (*tal*) gene, for example, controls tissue folding in *Drosophila* and encodes a ∼1.5 Kb transcript [23], whose putative ORFs are all extremely short. *Tal* was therefore initially classified as an ncRNA [24], but it has subsequently been shown that it is actually translated into multiple 11 aa peptides that fulfill the function of the gene [23]. With examples such as this that highlight the perils of dismissing even the shortest of putative ORFs, one wonders how many other presumed ncRNAs encode real albeit very short proteins.

#### ORF conservation

Given the problems of relying solely upon ORF size, an alternative approach to discriminating long ncRNAs from mRNAs is to assess putative ORFs for similarity to known proteins or protein domains, since such homology provides indirect evidence of function as an mRNA. Indeed, the vast majority of putative human ORFs without cross-species counterparts is likely to be random occurrences [25], and many studies of individual ncRNAs now cite a lack of ORF conservation to argue against function as an mRNA. Several tools and resources are available for such analysis, including BLASTX [26], rsCDS [27], Pfam [28], and SUPERFAMILY [29].

A few methods designed to detect ORF conservation can be used to distinguish ncRNAs from mRNAs on a transcriptome-wide scale. These comparative approaches include the programs CSTminer [30],[31] and CRITICA [32], both of which exploit the tendency for protein-coding sequences to favor synonymous base changes (i.e., changes that do not result in amino acid substitution) over non-synonymous ones, but are limited by the numbers of genomes available for comparison and the rapid evolution of many ncRNAs [33], making it difficult to detect orthologous sequences.

There are also other problems in using ORF conservation to identify protein-coding RNAs. First, these approaches are limited by the comprehensiveness and accuracy of current protein annotations. For instance, *Xist* was annotated as a protein-coding gene in public databases (Swiss-Prot accession: P27571) for almost fifteen years after its characterization as a functional ncRNA [21], which led to inadvertent misclassification by a computational pipeline in one recent study [34]. Second, some ncRNAs have evolved from protein-coding genes [35],[36], and so will retain remnant signatures of and homologies to mRNAs. Finally, particularly in less complex eukaryotes, such as yeast, absence of ORF conservation even with closely related species, does not guarantee an absence of function. Indeed, a recent study of orphan ORFs in *Saccharomyces cerevisiae* that had been initially annotated as spurious showed that many produced detectable transcripts and/or translated products [37].

#### Structural approaches

The approaches described above are primarily designed to identify mRNAs. Consequently, long ncRNAs are typically defined indirectly through an absence of mRNA-like characteristics. In contrast, a number of studies have used the presence of conserved predicted RNA secondary structure to identify ncRNAs imputed to have functional properties. These include the programs QRNA [38], RNAz [39], and EvoFOLD [40]. However, using these programs to classify transcripts as ncRNAs is likely to lead to significant false positive and false negative discoveries, since conserved secondary structures are also commonly found in mRNAs (especially 3′ UTRs), and functional ncRNAs may contain secondary or tertiary structures with non-canonical base interactions [41] that are not considered by structural prediction programs.

#### Experimental strategies

As well as computational methods, several experimental strategies have also been used to try to distinguish mRNAs and ncRNAs. For instance, in vitro translation assays have been performed in individual cases to test whether a putative ORF is translated into protein [23],[42],[43]. Positive translation results gives an indication that a transcript is an mRNA, but one needs to interpret such results with caution, since spurious ORFs have previously been translated in vitro and antibodies have even been generated against the resultant protein [44]. Meanwhile, negative translation results also tend to be inconclusive. Another experimental method is to assess whether a transcript is associated with polysomes [21], as would be expected for mRNAs that are actively translated, but again such a method is far from definitive.

#### Artifact filtering

Reliable classification of novel transcripts into mRNAs or ncRNAs is predicated on the assumption that they represent genuine, full-length transcripts. However, incomplete reverse transcription, internal priming of pre-mRNAs, and genomic contamination can all result in the generation of spurious or truncated transcripts, many of which are likely to masquerade as ncRNAs [45]. To help address this issue, various approaches have been used to filter out potential experimental artifacts. The FANTOM3 consortium, for instance, whittled down their original list of 34,030 ncRNAs to a shortlist of 3,652 confidently full-length ncRNAs by requiring that transcripts have stringent support at their 5′ and 3′ ends [5]. Another method excluded ncRNAs that mapped to the same genomic strand and locus as an mRNA, in the belief that such transcripts were likely to represent spuriously truncated mRNAs [18]. Notably, both these approaches are likely to filter out genuine ncRNAs, since not only are very long (>5 Kb) ncRNAs such as *Xist* and *Air* unable to be successfully captured as full-length transcripts using current cloning and sequencing approaches [34], but many genomic loci are also now known to harbor overlapping and/or interleaving mRNAs and ncRNAs on the same strand [13].

#### Combination strategies

Many of the strategies described above are complementary, and can be combined to good effect. For instance, CRITICA uses statistical techniques in addition to its comparative approach [32] and was the best-performing of ten bioinformatic methods used to discriminate ncRNAs and mRNAs from the FANTOM cDNA collection [20]. A number of other programs use sophisticated statistical approaches based on integrating a range of characteristic protein-coding signatures, including splice acceptor/donor sites, polyadenylation signals, ORF length, and sequence homology. For example, to discriminate coding regions, DIANA-EST employs a combination of artificial neural networks and statistical approaches [46], and ESTScan uses a hidden Markov model approach [47].

Two recently described tools, CPC and CONC, use supervised learning algorithms known as support vector machines to distinguish mRNAs from ncRNAs [48],[49]. These algorithms take into consideration multiple features such as peptide length, amino acid composition, protein homologs, secondary structure, and protein alignment information. Both showed high levels of accuracy when cross-validated against reference protein and ncRNA datasets, and are likely to represent the vanguard of future discrimination methods.

## Bifunctional RNAs and the False Dichotomy

Despite recent advances such as support vector machines to distinguish ncRNAs from mRNAs, large numbers of novel transcripts remain ambiguous and difficult to definitively categorize. CONC, for instance, estimated that ∼28,000 FANTOM cDNAs were ncRNAs, but >50% of these predictions fell outside the reliable range [48]. Are these ncRNAs or mRNAs? Currently, we cannot really say, but perhaps the question is itself flawed. After all, reports are now emerging of transcripts that can not only be translated into protein but also function independently as RNA, and the very existence of such bifunctional RNAs challenges the assumption that the RNA world can be neatly parsed between mutually exclusive protein-coding and noncoding categories.

The first report of a bifunctional RNA was the human *Steroid Receptor Activator* (*SRA*). Originally, *SRA* was characterized as an ncRNA, which functioned at the transcript level to co-activate steroid hormone receptors [43]. Remarkably, *SRA* transcripts have now been shown to also encode a functional protein (SRAP) [50], which appears to act antagonistically to SRA RNA at steroid hormone receptors [51]. This raises the intriguing possibility that bifunctional transcripts can negatively regulate their own functions, although just how such a process operates and is controlled requires further study.

Additional examples of bifunctional RNAs have also recently emerged. The *VegT* RNA has been known for many years to encode a protein needed to establish the primary germ layers in *Xenopus* [52]. However, *VegT* RNA has since been shown to also fulfill a separate structural role in the cytokeratin network of primordial germ cells [53]. In *Drosophila*, *Oskar* RNA was first characterized for its ability to be translated into one of two proteins important for oocyte development [54],[55]. Recently, it has been found that *Oskar* mRNA (specifically, its 3′ UTR) functions independently of the Oskar protein and is also essential for oogenesis [56]. Moreover, some 3′UTRs can regulate cell proliferation and differentiation in mammals, independently of their associated protein-coding sequences, with perturbations of this information in certain cancers [57]–[62]. There are also examples in bacteria. *SgrS* is a 227 nt transcript from *Escherichia coli* that functions to relieve the effects of glucose–phosphate stress. It functions as an RNA by base-pairing with the glucose transporter ptsG mRNA to negatively regulate its translation and stability [63] but also encodes a 43 aa protein, which acts to further reduce glucose uptake by inhibiting ptsG transporter activity [64]. In this way, the *SgrS* RNA functions through two distinct mechanisms to protect cells from glucose–phosphate stress. Moreover, these two functions appear to be physiologically redundant [64], which indicates that in some situations bifunctionality represents an inbuilt failsafe.

The number of documented cases of bifunctional RNAs is limited. However, as mentioned earlier, conserved secondary structures are commonly found in mRNAs, which suggests that bifunctional RNAs might be widespread. Indeed, it was recently predicted that in yeast as much as 5% of mRNAs function independently as RNA, and it was estimated that this proportion is likely to be significantly greater in higher eukaryotes [65]. To further confound this dichotomy, recent studies show that mRNAs that form duplex RNA structures within themselves, or with other antisense RNAs or pseudogenes, may be processed into endogenous siRNAs, therefore providing mRNAs with an additional fate [66]–[68]. Even synonymous sites in codons, often thought to be fully redundant, can encode additional subtle information. For instance, “silent” mutations in synonymous sites can affect both splicing and co-translational folding, and thereby alter protein function [69],[70]. This suggests that RNA carries much more *cis*- and *trans*-acting information than previously imagined, both within and beyond protein-coding sequences.

## Conclusions

As the number of protein-coding genes continues to be revised downward [25], there appears to be an ever-growing catalogue of ncRNAs. Nevertheless, there is an ongoing lack of clarity regarding the true number of ncRNAs within the genome. This is at least partly due to the inherent difficulties in discriminating ncRNAs from mRNAs and artifacts, especially amongst the thousands of long transcripts that defy categorization by even the most sophisticated of today's classification methods. This situation is further complicated by the emerging realization that the transcriptome may not consist of discrete separable species, but in reality comprise a series of overlapping clusters, of which many span large genomic regions [5],[71],[72], potentially comprising an information continuum. Looking ahead, we must also be prepared to cast off our historical biases toward what appears now to be an increasingly false dichotomy, and instead embrace the likelihood that RNA is a molecular multi-tasker, whose roles can simultaneously bridge both protein-coding and noncoding domains, and not only have more than one embedded function but also produce multiple products.
